# Imaging-based detection and prediction of radiation-induced cardiac toxicity: a narrative review

**DOI:** 10.1093/bjro/tzag008

**Published:** 2026-05-11

**Authors:** Keyur D Shah, Jun Zhou, Vanessa L Wildman, Justin Roper, Aparna H Kesarwala, Hania Al-Hallaq, Venkatesh L Murthy, Reshma Jagsi, Xiaofeng Yang

**Affiliations:** Department of Radiation Oncology, Winship Cancer Institute, Emory University, Atlanta, GA 30322, United States; Department of Radiation Oncology, Winship Cancer Institute, Emory University, Atlanta, GA 30322, United States; Department of Radiation Oncology, Winship Cancer Institute, Emory University, Atlanta, GA 30322, United States; Department of Radiation Oncology, Winship Cancer Institute, Emory University, Atlanta, GA 30322, United States; Department of Radiation Oncology, Winship Cancer Institute, Emory University, Atlanta, GA 30322, United States; Department of Radiation Oncology, Winship Cancer Institute, Emory University, Atlanta, GA 30322, United States; Division of Cardiovascular Medicine, Department of Internal Medicine, University of Michigan, Ann Arbor, MI 48109, United States; Department of Radiation Oncology, Winship Cancer Institute, Emory University, Atlanta, GA 30322, United States; Department of Radiation Oncology, Winship Cancer Institute, Emory University, Atlanta, GA 30322, United States; Department of Radiation and Cellular Oncology, The University of Chicago, IL 60673, United States

**Keywords:** radiation-induced cardiac toxicity, cardio-oncology, cardiac imaging, radiomics

## Abstract

Radiation therapy (RT) can lead to late-onset cardiac toxicity, especially in patients receiving treatment for breast, thoracic, or hematologic malignancies. Early identification of subclinical cardiac injury is essential to guide surveillance and intervention strategies. This narrative review synthesizes imaging-based evidence published between 2014 and 2025 on the detection and prediction of RT-induced cardiac toxicity. We screened 1535 studies and included 78 studies that employed echocardiography, cardiac magnetic resonance imaging (CMR), positron emission tomography (PET) and single-photon emission CT (SPECT), cardiac CT, or multimodal approaches. Echocardiographic strain imaging, particularly global longitudinal strain (GLS), consistently identified early subclinical dysfunction. CMR offered spatial and tissue-level characterization of myocardial fibrosis and inflammation, while PET/SPECT revealed perfusion and metabolic changes in irradiated myocardium. CT-based methods such as coronary artery calcium scoring and coronary CT angiography detected subclinical atherosclerosis with dose correlations. Several predictive models integrating imaging, dose, and clinical data demonstrated strong performance in identifying high-risk patients. Despite encouraging results from such models, variability in timing and endpoint selection limits clinical adoption. Standardized imaging protocols and prospective validation of predictive models are urgently needed. Imaging biomarkers may help tailor follow-up strategies to individual risk and guide the design of future cardio-oncology trials and radiation treatment planning strategies.

## Introduction

Radiation therapy (RT) remains a cornerstone in the curative treatment of thoracic malignancies such as breast, lung, lymphoma, and esophageal cancers.^1–5^ As cancer survival improves, delayed cardiac injury from radiation has become a pressing clinical issue.^6^^,^^7^ Radiation-induced heart disease (RIHD) encompasses a wide range of clinical and subclinical conditions, such as accelerated atherosclerosis, myocardial fibrosis, conduction abnormalities, valvular dysfunction, and heart failure.^8–10^

Large population-based studies have consistently demonstrated a dose-dependent increase in the risk of major adverse cardiac events, with no definitive threshold below which the heart is entirely safe.^11^^,^^12^ For instance, each 1 Gy increase in mean whole-heart dose has been associated with a 7%-16% increase in the relative risk of coronary events.^13^^,^^14^ While modern techniques such as intensity-modulated radiotherapy (IMRT), proton therapy, and deep inspiration breath-hold (DIBH) have reduced incidental cardiac exposure,^15^ even low to moderate doses may induce subclinical myocardial injury that precedes overt cardiac events.^16–18^

Conventional tools for monitoring cardiac function, such as left ventricular ejection fraction (LVEF), lack sensitivity for detecting early injury.^19–21^ In contrast, multimodality cardiovascular imaging techniques, including speckle-tracking echocardiography (STE), cardiac magnetic resonance imaging (CMR), nuclear imaging, and cardiac CT offer more sensitive and quantitative endpoints. These imaging biomarkers are increasingly used for risk stratification, early diagnosis, and longitudinal surveillance.^22–25^

More recently, artificial intelligence (AI), machine learning, and radiomics-based methods have emerged as promising tools. These approaches integrate imaging, dosimetry, and clinical data to predict radiation-associated adverse cardiac events.^24^^,^^26^ Cardiotoxicity refers to short- and long-term damage to the heart due to cancer therapeutics such as RT or chemotherapy and includes both subclinical and clinical outcomes. These predictive models offer the potential for precision surveillance, enabling clinicians to identify high-risk patients for more intensive monitoring or early cardioprotective interventions.

In this narrative review, literature from 2014 to 2025 on imaging-based detection and prediction of radiation-induced cardiotoxicity has been synthesized. The capabilities and limitations of different imaging modalities are reviewed, followed by a highlight of predictive models for cardiotoxicity and a discussion of their potential for clinical translation. Finally, key research gaps are identified, and future directions are offered to advance precision cardio-oncology. To our knowledge, this is the first comprehensive review integrating multimodality imaging and predictive modeling in the context of RT-induced cardiac toxicity.

## Study selection and scope

To identify relevant studies, a structured literature search was conducted using PubMed and Scopus using the following terms: (“radiation therapy” OR “radiotherapy”) AND (“cardiac toxicity” OR “cardiotoxicity” OR “myocardial fibrosis” OR “coronary artery disease”) AND (“imaging” OR “echocardiography” OR “CMR” OR “MRI” OR “CT” OR “PET” OR “SPECT”). Searches were limited to studies published between January 1, 2014 and June 12, 2025, involving human subjects and published in English.

The combined database search yielded 1535 articles, which were manually screened for relevance based on title, abstract, and full text. Studies were included if they (1) evaluated cardiac structure or function using imaging (echocardiography, CMR, CT, PET, or SPECT), (2) involved patients who had received RT, and (3) reported imaging-based endpoints related to cardiac toxicity such as fibrosis, perfusion defects, coronary calcification, or changes in strain/LVEF. Studies that additionally reported or correlated radiation dose metrics (eg, mean heart dose (MHD), V5, LAD dose) were noted but not required. After full-text screening, 76 studies were included and categorized by imaging modality and analytic strategy. Additionally, 2 studies were identified through independent citation analysis and included based on relevance.

Studies were excluded if they focused solely on chemotherapy-induced cardiotoxicity, used biomarkers without imaging, were preclinical/animal studies, or described radiation planning without follow-up imaging. Studies involving both chemotherapy and RT were included only if RT-specific cardiac imaging outcomes were clearly reported. Most excluded studies were either biomarker-only, preclinical models, or focused solely on chemotherapy-induced toxicity. Additionally, while several studies have characterized dose-response relationships independently of imaging, scope was limited to studies where imaging served as a primary endpoint.

**Figure 1:**
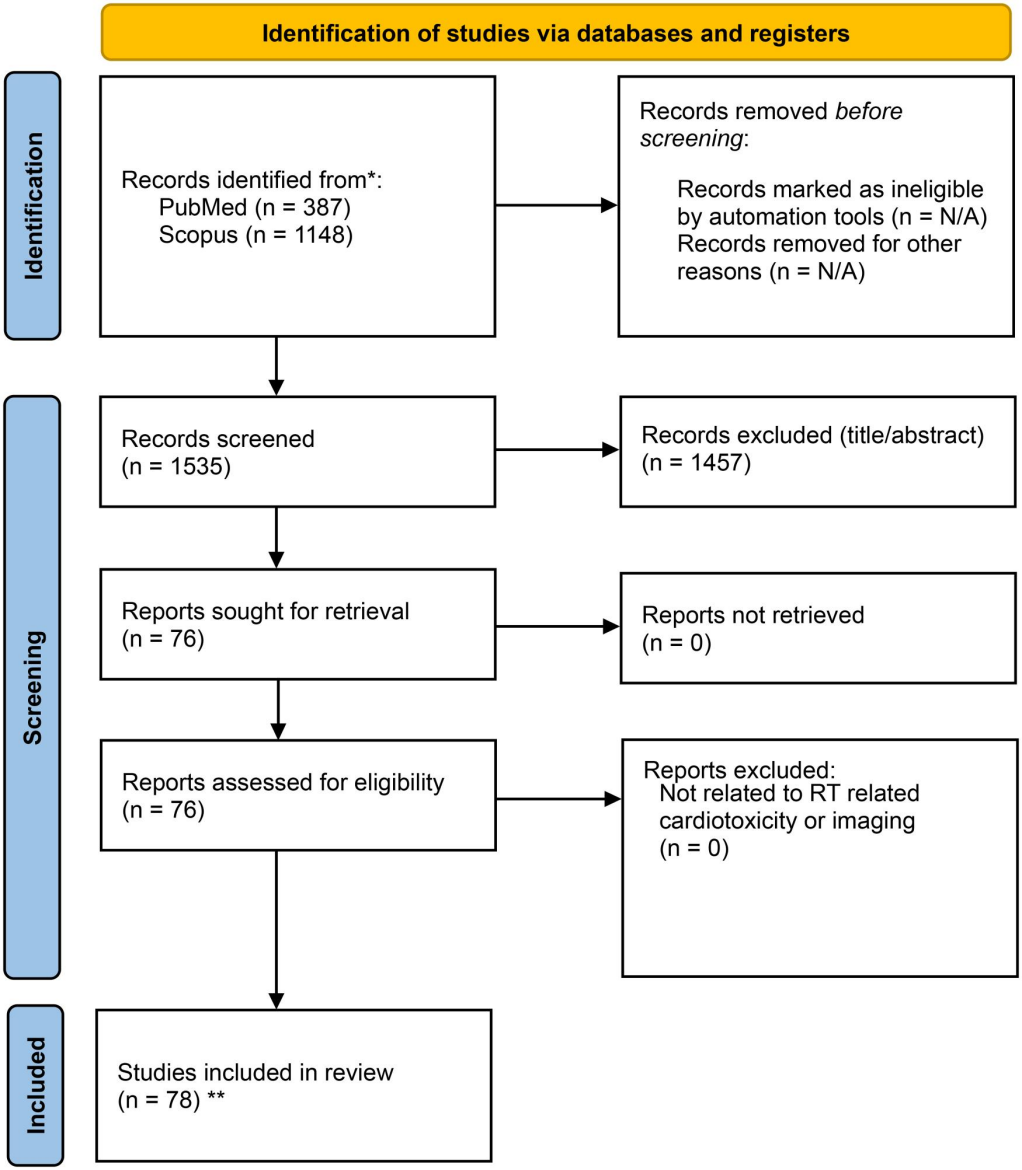
PRISMA flow diagram outlining the systematic search, screening, and selection process for studies included in the review on imaging-based assessment of radiation-induced cardiac toxicity. The diagram highlights the identification of relevant studies from databases, the filtering process, reasons for exclusion, and the final number of 78 studies included in the review. **n = 2 studies were added after independent citation analysis.

Eligible studies were categorized by imaging modality and study design. Studies using AI, radiomics, or other predictive modeling techniques were flagged separately and summarized in a dedicated section. A PRISMA-style flow diagram is included to illustrate the selection process in Figure 1. The literature search and initial screening were conducted by KDS, a medical physicist currently in clinical residency training with approximately 8 years of research experience in medical imaging analysis and RT-related imaging outcomes. Study selection and data extraction were subsequently reviewed by the co-authors to ensure consistency and relevance to the scope of the review.

## Imaging modalities used for the detection and characterization of radiation-induced cardiac toxicity

A wide range of imaging modalities have been employed to detect and characterize radiation-induced cardiac toxicity. These span functional, anatomical, and molecular approaches, each offering unique strengths depending on the clinical context and timing of assessment. In this section, 71 of the 78 included studies are categorized based on the primary imaging modality used for cardiac evaluation following RT.

### Echocardiography

Echocardiography remains the most widely used modality for cardiac surveillance in patients undergoing RT, owing to its accessibility, cost-effectiveness, and ability to provide real-time functional assessment of cardiac structure and function.^27–29^ LVEF measured from M-mode or 2D imaging is the most widely used clinical metric, but may miss early signs of cardiac dysfunction^30^ and, particularly in patients with normal or near-normal LVEF.^31–33^

In recent years, advanced echocardiographic techniques such as 2D STE, the most widely used form of myocardial strain imaging, have enabled the assessment of myocardial deformation via global longitudinal strain (GLS), offering earlier detection of subclinical dysfunction.^34^ Across multiple studies, a consistent pattern of GLS decline has been observed within weeks to months following RT, even in asymptomatic patients with preserved LVEF.^35–37^ Several of these studies have also demonstrated a dose-response relationship, with more pronounced strain reductions in patients receiving higher MHDs or elevated doses to substructures such as the left anterior descending artery (LAD).^36^^,^^38^^,^^39^

Long-term studies in survivors of childhood and adolescent cancers have demonstrated persistent abnormalities in strain imaging decades after treatment, often without significant changes in LVEF.^33^^,^^40^^,^^41^ These findings reinforce the value of GLS as a surveillance biomarker in both early- and late-phase cardiotoxicity. Furthermore, protocol-driven multicenter efforts have confirmed the feasibility and reproducibility of strain-based assessment in large-scale survivorship studies.^42^

Beyond GLS of the LV, additional echocardiographic metrics such as right ventricular free wall strain (RVFWS), tricuspid annular plane systolic excursion (TAPSE), and diastolic function parameters (eg, E/e′) have been explored. Some studies have shown RVFWS decline in association with right heart radiation exposure,^36^^,^^43^ and others demonstrated that diastolic dysfunction may serve as an earlier indicator than systolic decline, particularly in patients treated with concurrent therapies like trastuzumab or aromatase inhibitors.^44^ Taken together, echocardiography, especially with incorporation of strain imaging, provides a sensitive and noninvasive tool for the early detection and longitudinal monitoring of radiation-associated cardiac dysfunction. Reported sensitivities of GLS for detecting subclinical cardiotoxicity range from 80% to 90%, though specificity of 80% depending on population and thresholds used.^45–47^ Representative studies using echocardiography are summarized in Table 1.

**Table 1:** Summary of studies assessing cardiac function using echocardiography in patients receiving RT.

Author (year)	Modality	Cancer type	Sample size (*n*)	Timepoint(s)	Key findings
Armstrong et al. (2015)^32^	Echo	Childhood cancers	1820	Median 23 years post-treatment	GLS abnormal in 28% with preserved EF; strongly associated with RT dose and anthracycline exposure
Aula et al. (2018)^48^	Echo	Breast	73	Pre-RT, end-RT	TAPSE/cIBS decline associated with reductions in TGF-β1 and PDGF; biomarker-linked echocardiographic changes
Aula et al. (2020)^49^	Echo	Breast	63	Pre-, post-RT, 3 years	ST2 increase linked with GLS/LVEF decline at 3 years; potential biomarker for long-term toxicity
Berlin et al. (2025)^50^	Echo	Breast	303	Pre-RT, longitudinal follow-up (median 5.1 years)	Circumferential and longitudinal strain worsened over time despite stable/improved LVEF; maximum LAD dose associated with deterioration in systolic and diastolic function
Bonzano et al. (2018)^51^	Echo	Breast (HER2+)	52	Median 5 years	No significant LVEF decline across HRT schemes; concurrent trastuzumab was not associated with increased toxicity
Cao et al. (2015)^44^	Echo	Breast (HER2+)	143	Pre-, post-, 3 and 6 months	Diastolic dysfunction more sensitive than LVEF; associated with heart DVH metrics during concurrent RT + trastuzumab
Christiansen et al. (2016)^33^	Echo	Childhood lymphoma/ALL	191	Cross-sectional	GLS impaired in 28% despite normal EF; associated with mediastinal RT and anthracycline dose
Ekici et al. (2016)^52^	Echo	Thoracic (lung + breast)	35	Pre-RT, 1-month post-RT	Strain rate imaging detected early LV and RV strain reduction after RT despite unchanged LVEF and biomarkers
Fourati et al. (2022)^53^	Echo	Breast	103	Pre-RT, 3, 6, 12 months	Segmental GLS decline associated with higher LV segment radiation dose; anteroseptal and apical regions most affected
Hassan et al. (2023)^54^	Echo	Breast (proton RT)	70	Pre-, 4 weeks, 2 months	No significant change in GLS or biomarkers overall, but hypertensive patients showed GLS decline
Liu et al (2024)^36^	Echo	Thoracic (mixed)	40	Pre-, mid-, post-RT, follow-up	2D-STE detected early systolic dysfunction; GLS and RVFWLS decline correlated with MHD and V5
Lo et al (2017)^37^	Echo	Breast (left-sided)	40	Pre-, 6 weeks	Segmental strain decline most notable in apical regions; correlated with local dose
Locquet et al (2022)^55^	Echo	Breast	186	Pre-RT, 6 months	GLS-based subclinical LV dysfunction in 14.4%; higher whole-heart and LV doses associated with GLS decline; LV-V5 strongest predictor
Marrazzo et al (2024)^56^	Echo	Breast (SAFE trial)	39	Post-RT	Subclinical damage (≥10% GLS/EF drop) most frequent in placebo arm; MHD did not differ between groups
Merkx et al (2021)^42^	Echo	Childhood cancers	1679	Cross-sectional	Feasible and reproducible Echo protocol (GLS, GCS, RVFWS); supports Echo as a surveillance tool in survivors
Nack et al (2020)^57^	Echo	Breast	110	Post-RT	New myocardial and valvular abnormalities more frequent with left-sided RT and MHD >2 Gy
Rihackova et al (2025)^41^	Echo	Lymphoma	167	Median 10 years	GLS declined in survivors with low-normal EF; proposed as follow-up trigger in survivors
Skyttä et al (2015)^43^	Echo	Breast (left-sided)	60	Pre-, post-RT	TAPSE and diastolic function declined more with concurrent aromatase inhibitor use
Toma et al (2023)^38^	Echo	Breast (HER2+)	68	Pre-, post-RT	EF drop >5% linked to high LAD dose; baseline ECG changes and anti-HER2 therapy increased risk
Trivedi et al (2019)^58^	Echo	Breast (left-sided)	40	Baseline, 6 weeks, 12 months	Persistent GLS reduction despite stable LVEF; strain abnormalities detectable early after RT
Tuohinen et al (2022)^59^	Echo	Breast	99	Baseline, post-RT, 3 years	Decline in septal cyclic variation of integrated backscatter associated with LAD radiation dose; GLS and LVEF unchanged
Walker et al (2019)^35^	Echo	Breast	79	Pre-, 6 months	GLS decline >10% associated with MHD, LV V20; effect attenuated after adjusting for endocrine therapy
Walker et al (2020)^39^	Echo	Breast	64	Pre-, 6 months	Endocardial GLS most sensitive for detecting segmental strain loss in high-dose LAD/apical regions
Yu et al (2016)^60^	Echo	Childhood, adolescent, and young adult	134	Cross-sectional	GLS more frequently abnormal than LVEF; worsened by prior mediastinal RT
Yu et al (2019)^40^	Echo	Breast (HER2+)	47	Pre-chemo, Pre-RT, Post-RT, 6 months	No significant changes in GLS, circumferential/radial strain, or diastolic function post-RT; minimal EF drop; MHD not associated with Echo findings

Abbreviations: 2D-STE = 2D speckle-tracking echocardiography; cIBS = calibrated integrated backscatter; DVH = dose-volume histogram; ECG = electrocardiogram; Echo = echocardiography; EF = ejection fraction; GCS = global circumferential strain; GLS = global longitudinal strain; Gy = gray; HER = human epidermal growth factor receptor; HRT = hypofractionated radiotherapy; LAD = left anterior descending artery; LVEF = left ventricular ejection fraction; MHD = mean heart dose; PDGF = platelet-derived growth factor; RT = radiotherapy; RVFWS = right ventricle free wall strain; RVFWLS = right ventricle free-wall longitudinal strain; TAPSE = tricuspid annular plane systolic excursion; TGF-β1 = transforming growth factor-beta 1.

### Cardiac magnetic resonance imaging

Cardiac magnetic resonance imaging enables detailed tissue characterization and has emerged as a valuable tool for detecting radiation-induced myocardial fibrosis, edema, and remodeling. Across 10 studies, CMR endpoints such as native and post-contrast T1, T2, extracellular volume (ECV) fraction, and late gadolinium enhancement (LGE) were used to evaluate subclinical cardiac injury following RT.

Breast cancer cohorts were the most studied, typically receiving contemporary left-sided RT with low mean whole-heart doses. Speers et al observed post-RT alterations in T1 mapping and RV ejection fraction, although these changes were not dose-correlated, and T2 or LV function remained stable.^61^ Lamy et al found a persistent decrease in LV GLS on CMR at 2 years post-RT in 16.6% of participants, which correlated with higher whole-heart and LV doses.^62^

In esophageal cancer cohorts, higher radiation doses to the LV allowed clearer demonstration of dose-response effects. In Umezawa et al, post-chemo RT (CRT) CMR revealed fibrosis in 43% of patients, defined by LGE and/or T1 elevation within high-dose regions (eg, LV V45 > 2.1%).^63^ de Groot et al conducted a dose-mapping study correlating segment-wise radiation dose with ECV elevation, showing a linear increase of 0.136% per Gy in long-term survivors.^64^

Taken together, these studies affirm the value of CMR in identifying both early and late myocardial alterations, especially fibrosis, with several demonstrating spatial concordance between imaging changes and irradiated regions. However, variability in scan timing, endpoints, and concurrent therapies (eg, anthracyclines) complicate interpretation. While not routinely implemented in clinical follow-up, CMR-based endpoints such as LGE or ECV may hold promise as early surrogate biomarkers in future cardio-oncology trials. A summary of studies evaluating RT-associated cardiac injury using cardiac MRI, including fibrosis, strain changes, and tissue characterization endpoints is provided in Table 2.

**Table 2:** Summary of studies assessing cardiac function using cardiac magnetic resonance imaging (CMR) in patients receiving RT.

Author (year)	Modality	Cancer type	Sample size (*n*)	Timepoint(s)	Key findings
de Groot et al (2021)^64^	CMR	Esophageal	33	∼68 months post-RT (nCRT) vs 122 months (control)	Higher global ECV in nCRT group; linear ECV increase with segment dose (∼0.136%/Gy); no EF/LGE differences
de Ville de Goyet et al (2015)^65^	CMR	Childhood cancers	81	Baseline and annual follow-up	Increased left atrial volume and myocardial scarring correlated with radiation dose
Huang et al (2016)^66^	CMR	Thoracic (mixed)	7	Post-RT	LA scar enhancement on LGE-MRI correlated with local radiation dose
Lamy et al (2025)^62^	CMR	Breast	138	Baseline, 6 months, 24 months	Persistent GLS declines in 16.6%; higher WH and LV doses linked to GLS decline; subtle LV remodeling without symptoms
Ricco et al (2020)^67^	CMR	Thoracic (mixed)	28	Mean 46 months post-RT	No association between cardiac dose and LGE or T1 values; feasibility study of CMR fibrosis markers
Speers et al (2022)^61^	CMR	Breast (left-sided)	51	Pre-RT, End-RT, 3 months post-RT	T1 and RVEF changed post-RT but not dose-dependent; MHD ∼2 Gy; IL-6 and troponin showed weak associations with dose
Tian et al (2023)^68^	CMR	Thoracic (mixed)	15	Baseline, 3 months, 6 months	Native T1/T2 increased in irradiated myocardium despite stable LVEF
Thavendiranathan et al (2023)^69^	CMR	Breast (HER2+)	136	Baseline, mid-treatment, every 3 months	Transient changes in T1, T2, ECV; associated with BNP and remodeling but not CTRCD; no LVEF/strain impact
Umezawa et al (2014)^70^	CMR	Esophageal	24	∼24 months post-RT	LGE observed in segments receiving ≥40 Gy; none in unirradiated segments; fibrosis mainly mid-myocardial
Umezawa et al (2023)^63^	CMR	Esophageal	23	Pre-CRT, 6 months post-CRT	RT-induced myocardial damage detected in 10/23; LV V45 ≥ 2.1% predicted damage; linked to later cardiac events

Abbreviations: CMR = cardiac magnetic resonance imaging; CTRCD = cancer therapy-related cardiac dysfunction; ECV = extracellular volume; EF = ejection fraction; GLS = global longitudinal strain; Gy = gray; HER = human epidermal growth factor receptor; LGE = late gadolinium enhancement; LV = left ventricle; LVEF = left ventricular ejection fraction; MHD = mean heart dose; nCRT = neoadjuvant chemoradiation; RT = radiotherapy; RVEF = right ventricular ejection fraction; WH = whole heart.

### PET and SPECT

Positron emission tomography (PET) and single-photon emission CT (SPECT) have emerged as sensitive imaging modalities for evaluating myocardial perfusion and metabolism which may be early signs of radiation-induced cardiac toxicity. Compared to structural imaging, these techniques uniquely provide functional insight, enabling detection of subclinical ischemia and perfusion defects.

Among PET-based studies, 13N-ammonia PET has demonstrated feasibility and the ability to detect declines in myocardial flow reserve (MFR), a sensitive quantitative marker of blood flow to the myocardium, within a year post-RT, particularly in myocardial segments exposed to higher radiation doses.^71^ In contrast, a separate 13N-ammonia PET study using breath-hold adapted RT found no significant differences in rest or stress myocardial blood flow (MBF) between irradiated and non-irradiated myocardium, suggesting that modern techniques may mitigate early perfusion changes.^72^

Single-photon emission CT studies, especially with 99mTc-MIBI, have also shown early evidence of myocardial wall motion abnormalities and perfusion defects during the course of RT, specifically after a cumulative dose of 40 Gy, with these changes correlated to dose-volume metrics.^73^ Longitudinal SPECT data in breast cancer survivors indicate that perfusion defects may persist even 6 months post-RT, with risk potentially modifiable through breath-hold techniques.^74^ A randomized trial (NCT00581256) demonstrated that IMRT with DIBH was associated with a small but statistically significant preservation of LVEF compared to standard free-breathing 3D conformal RT (3D-CRT) in patients with node-positive left-sided breast cancer.^75^ While the primary endpoint, perfusion defects on SPECT, did not differ between groups, the finding highlights the potential value of incorporating functional measures like LVEF into future trials, and reinforces the importance of minimizing cardiac dose even when baseline exposure is relatively low. Additionally, factors such as BMI, inflammation markers, and history of left-sided RT have been linked to perfusion abnormalities detected via SPECT.^76^

PET and SPECT nuclear imaging modalities provide early, dose-sensitive markers of RT-associated metabolic changes and myocardial injury as summarized in Table 3. The detection of early perfusion and metabolic changes highlights the value of PET and SPECT in high-risk patients, where timely intervention may modify disease progression.

**Table 3:** Summary of studies assessing cardiac function using nuclear imaging (PET or SPECT) in patients receiving RT.

Author (year)	Modality	Cancer type	Sample size (*n*)	Timepoint(s)	Key findings
Jagsi et al (2018)^75^	SPECT	Breast cancer	54	Baseline and 1-year post-RT	RCT comparing IMRT-DIBH vs standard 3D-CRT; no difference in LAD perfusion defects; IMRT-DIBH preserved LVEF; lower LV and heart doses observed with IMRT-DIBH
Matsuo et al (2023)^77^	PET/CT	Esophageal cancer	187	Baseline and up to 2 years	CAC progressed in all groups, esp. with middle/lower chest RT and pre-existing CAC
Melichar et al (2014)^76^	SPECT	Breast cancer	181	Single timepoint	Perfusion defects (7%) linked to BMI, inflammation, left-sided RT
Nehmeh et al (2020)^71^	PET	Breast cancer	10	Pre-RT and ∼13 months post-RT	Feasibility of 13 N-ammonia PET shown; MFR decreased in 50% of patients, associated with high-dose regions
Ning et al (2017)^78^	PET/CT	NSCLC	201	Post-RT (median 8.9 months)	HV35 > 10% strongly predicted pericardial effusion; validated in independent cohort
Rasmussen et al (2021)^72^	PET	Breast cancer	20	∼7 years post-RT	No MBF changes between irradiated vs non-irradiated myocardium; 1 patient had defect
Wang et al (2023)^79^	SPECT	Breast cancer (left-sided)	61	Pre-RT and 12 months post-RT	New myocardial perfusion defects associated with higher LV segment radiation doses
Zakem et al (2023)^80^	PET	Esophageal cancer	51	Pre- and post-RT PET	Dose-response observed between cardiac radiation dose and increased FDG uptake; SUV changes predicted overall survival
Zellars et al (2014)^74^	SPECT	Breast cancer	43	Pre-RT and 6 months post-RT	ABC did not reduce perfusion defects post-RT on SPECT
Zhang et al (2015)^73^	SPECT	Esophageal cancer	18	Pre-RT and during RT (40 Gy)	SPECT detected early myocardial injury and perfusion defects correlated with high-dose volumes
Zyromska et al (2018)^81^	PET/CT	Breast cancer	15	Pre-RT, 2 and 8 months post-RT	15O-H₂O PET detected reduced myocardial blood flow in LAD territory after RT; changes correlated with LAD dose

Abbreviations: 3D-CRT = 3D conformal radiotherapy; ABC = active breathing coordinator; BMI = body mass index; CAC = coronary artery calcification; DIBH = deep inspiration breath-′hold; Gy = gray; HV = heart volume; IMRT = intensity-modulated radiotherapy; LAD = left anterior descending artery; LV = left ventricle; LVEF = left ventricle ejection fraction; MBF = myocardial blood flow; MFR = myocardial flow reserve; NSCLC = non-small cell lung cancer; PET = positron emission tomography; RCT = randomized clinical trial; RT = radiotherapy; SPECT = single-photon emission CT.

### Cardiac CT and coronary CT angiography

Cardiac CT, particularly coronary artery calcium (CAC) scoring and coronary CT angiography (CCTA), offers detailed anatomical visualization of coronary artery disease (CAD) and has become increasingly important for long-term surveillance after thoracic RT. Across numerous studies, CT-based measures consistently demonstrate a dose- and laterality-dependent progression of coronary calcification post-RT. For instance, Lai et al^82^ observed accelerated CAC progression in patients with breast cancer, respectively, with greater increases linked to left-sided RT or middle-lower chest irradiation. Studies also highlight that even modest increases in CAC or calcification burden, for example, when more than 10% of heart volume receives 35 Gy or more (eg, HV35 > 10%) are predictive of future cardiac events, including pericardial effusion and myocardial infarction.^83^

Coronary CT angiography adds an additional layer of granularity by characterizing plaque composition, stenosis severity, and vessel involvement. Studies in lymphoma and breast cancer survivors revealed higher rates of multi-vessel disease, proximally located plaques, and severe nonostial stenoses compared to matched controls.^56^^,^^84^^,^^85^ Importantly, baseline CT parameters such as coronary stenosis and CT-derived fractional flow reserve (CT-FFR) have also been linked to future development of cancer therapy-related cardiac dysfunction (CTRCD).^86^

Notably, even among asymptomatic patients, cardiac CT has identified high-risk anatomical patterns warranting closer monitoring or intervention.^87^^,^^88^ These findings support the growing role of cardiac CT in risk stratification, early diagnosis, and integration into RT planning, particularly for patients with thoracic malignancies or preexisting cardiovascular risk. A structured overview of CT-based studies, including CAC scoring and CT angiography, is presented in Table 4.

**Table 4:** Summary of studies assessing coronary or cardiac changes using cardiac CT or CT angiography in patients receiving RT.

Author (year)	Modality	Cancer type	Sample size (*n*)	Timepoint(s)	Key findings
Barcellini et al (2024)^89^	CT (CIRT planning + 4D CT)	Cardiac/para-cardiac tumors	16	During and up to 6 months post-CIRT	No significant cardiac biomarker or echo changes; CIRT appears cardioprotective
Capra et al (2020)^90^	CT (contrast-enhanced ECV)	Esophageal cancer	21	Baseline, ∼35 days, ∼420 days post-RT	CT-derived myocardial ECV increased after RT and correlated with radiation dose
Girinsky et al (2014)^91^	CT angiography	Hodgkin lymphoma	179	Median 9.5 years post-treatment	CCTA detected CAD in 26%; radiation dose and conventional risk factors predictive
Honaryar et al (2022)^92^	CT (CAC)	Breast cancer	101	Baseline and 2 years post-RT	CAC progression associated with LV dose and LAD exposure
Krug et al (2025)^93^	CT (CAC + CTCA)	Breast cancer	75	∼11 years post-RT	CAC and CAD prevalence similar to matched controls; risk driven by CV factors rather than RT dose
Lai et al (2021)^82^	CT (CAC)	Breast cancer	94	Pre- and post-RT	Higher CAC progression in BC patients vs controls; left-sided RT and RCA dose linked to CAC increase
Lee et al (2019)^94^	CT	Breast cancer	3489	Long-term follow-up	RT and anthracycline both increased risk of heart failure and CAD
Mast et al (2016)^95^	CT (CAC)	Breast cancer	99	Pre-RT and 3 years post-RT	Breath-hold reduced CAC increase; LAD-RCA score differences seen in left-sided RT
Polomski et al (2025)^96^	CT angiography	Various cancers	312	Post-treatment (age-matched controls)	Cancer survivors, especially <60 years, had higher CAD prevalence; RT linked to increased risk
Puckett et al (2021)^97^	CT (CAC), EKG, Echo	Breast cancer	201	∼11.5 years post-treatment	High CVD incidence even 10+ years after treatment; each modality contributed uniquely to detection
Refsgaard et al (2025)^98^	CT (CAC from planning CT)	Breast cancer	3355	Up to 10 years post-RT	Agatston score predicted CAD; dose-response seen for MHD in low AS group
Tagami et al (2021)^85^	CT angiography	Breast cancer	94	Post-RT	Higher CAD incidence in LBC vs RBC; mean LAD dose correlated with coronary disease
Takada et al (2025)^99^	Cardiac CT	Esophageal cancer	41	Baseline (pre-RT)	44% had significant CAD before RT; suggests value of cardiac CT in RT planning
Takx et al (2017)^100^	CT (CAC)	Breast cancer	333	∼2 years post-RT	No increase in coronary calcium burden compared with non-RT patients
Tu et al (2024)^86^	CT angiography	Breast cancer	139	Pre- and post-treatment	Post-treatment CT showed CT-FFR ↓, PCAT CT ↑, ECV ↑; baseline LAD/RCA stenosis predicted CTRCD
van Rosendael et al (2017)^84^	CT angiography	Lymphoma	79	Median 19 years post-treatment	Irradiated patients had more proximal, severe CAD than matched controls
Wang et al (2022)^83^	CT (CAC)	NSCLC	109	Baseline (planning CT)	Baseline coronary calcification predicted post-RT cardiac events; high calcification = higher risk
Wethal et al (2014)^101^	CT (CAC)	Hodgkin lymphoma	43	FU-1 (5-13 years), FU-2 (18-27 years)	Radiation-associated atherosclerosis correlated with cholesterol levels and more prevalent than in non-irradiated controls

Abbreviations: BC = breast cancer; CAC = coronary artery calcification; CAD = coronary artery disease; CCTA = coronary CT angiography; CIRT = carbon ion radiotherapy; CT-FFR = CT-derived fractional flow reserve; ECV = extracellular volume; EKG = electrocardiogram; FU = follow-up; LAD = left anterior descending artery; LBC = left breast cancer; MHD = mean heart dose; NSCLC = non-small cell lung cancer; PCAT = pericoronary adipose tissue; RCA = right coronary artery; RBC = right breast cancer; RT = radiotherapy.

### Multimodality and integrative approaches

An increasing number of studies are adopting multimodal approaches that combine multiple imaging techniques or integrate imaging with electrocardiography, serum biomarkers, or radiation dosimetry to detect and characterize radiation-induced cardiac toxicity. These approaches provide complementary insights across structural, functional, and metabolic domains, often revealing early signs of injury not captured by a single modality.

For example, Moisander et al used echocardiography, ECG, and cardiac MRI (CMR) alongside segmental dosimetry to detect diffuse myocardial fibrosis up to 6 years post-RT, with changes localized to high-dose cardiac regions.^102^ Similarly, Ylänen et al applied real-time 3D echocardiography (RT-3DE) and CMR in childhood cancer survivors, identifying subtle ventricular dyssynchrony and reduced LVEF that standard echo might miss, particularly in those exposed to both anthracyclines and RT.^103^ Finally, the large-scale study by Bachir et al analyzed long-term follow-up from the HERA trial to evaluate whether the addition of RT to trastuzumab with left-sided RT, right-sided RT, or no RT, there were no significant differences in LVEF decline or cardiovascular event rates, suggesting that RT did not significantly exacerbate cardiotoxicity, regardless of laterality, when added to trastuzumab.^104^

These studies highlight the value of integrative imaging strategies in providing a more nuanced and sensitive assessment of cardiotoxicity, especially in settings where cumulative or modality-specific risk is high. Table 5 compiles studies using multimodal imaging strategies, including echo, CMR, CT, ECG, and biomarkers to assess composite measures of radiation-induced cardiac toxicity.

**Table 5:** Summary of studies using multimodal imaging approaches to assess radiation-induced cardiac toxicity.

Author (year)	Modality	Cancer type	Sample size (*n*)	Timepoint(s)	Key findings
Bachir et al (2022)^104^	Echo + clinical endpoints	Breast cancer (HER2+)	3321	Median 11 years follow-up	RT did not significantly increase risk of LVEF decline or cardiac events in trastuzumab-treated patients
Barcellini et al (2024)^89^	CT + Echo + ECG + biomarkers	Cardiac/para-cardiac tumors	16	During and 6-month post-CIRT	CIRT showed no significant biomarker or echo/ECG changes; suggests cardioprotective potential
Bergom et al (2020)^105^	CMR + RT dosimetry	Breast	20	Median 8.3 years post-RT	No abnormal LGE or EF decline; ventricular mean dose correlated with increased LV mass suggesting subclinical remodeling
Heggemann et al (2015)^106^	Echo + MRI	Breast	49	Pre-, 6, 12, 24 months	Minimal persistent changes; transient strain and EF reductions observed
Krug et al (2024)^107^	Echo + CMR + CTCA + biomarkers	Breast	76	∼11 years post-RT	Minimal regional strain changes; normal global function and low CAD prevalence after isolated RT
Moisander et al (2023)^102^	Echo + ECG + CMR + dosimetry	Breast cancer	30	Baseline, post-RT, 3-year and 6-year follow-up	Progressive diffuse fibrosis markers in Echo/CMR/ECG; regional changes correlated with segmental dose
Ylänen et al (2014)^103^	Echo + CMR	Childhood cancers	71	Cross-sectional	RT-3DE identified more LV function abnormalities than standard Echo; dyssynchrony metrics linked with RT

Abbreviations: CIRT = carbon ion radiotherapy; CMR = cardiac magnetic resonance imaging; ECG = electrocardiogram; Echo = echocardiography; EF = ejection fraction; HER2 = human epidermal growth factor receptor 2; LVEF = left ventricular ejection fraction; LV = left ventricle; MRI = magnetic resonance imaging; RT = radiotherapy; RT-3DE = radiation therapy-3D echocardiography.

## Image-based predictive models for radiation-induced cardiotoxicity

Several recent studies have developed predictive models, ranging from traditional risk scores to machine and deep learning frameworks, to identify patients at risk of radiation-induced cardiac toxicity using imaging, dosimetric, and patient-specific clinical variables. These models span a range of methods, including Cox regression, logistic regression, and radiomics-based approaches. Most focus on patients with breast or thoracic malignancies, where cardiac exposure during RT is more prevalent.

Notably, multiple studies have employed multi-modal machine learning approaches. Choi et al developed a deep convolutional neural network incorporating CT, dose distributions, and auto-segmented heart substructures to predict acute coronary events in patients with breast cancer, achieving an area under the curve (AUC) of 0.94 in cross-validation.^108^ Long et al used pretreatment CT radiomics features extracted from 10 cardiovascular structures to build a support vector machine classifier that predicted post-RT cardiac dysfunction in thoracic malignancies, reporting an accuracy of 0.88.^109^

Radiomics models have also shown promise in assessing pre-existing or evolving cardiac vulnerability. Choi et al trained a radiomics model on pretreatment FDG-PET/CT scans to classify abnormal cardiac FDG uptake patterns, potentially serving as a baseline risk stratification tool.^110^ Cho et al demonstrated that elevated left ventricular SUV on post-treatment FDG-PET, alongside MHD, independently predicted late cardiac events in patients with non-small cell lung cancer (NSCLC) using multivariable Cox regression analysis.^111^

These predictive models represent an important shift toward personalized surveillance and risk-adaptive management for patients undergoing thoracic radiation. A structured summary of these studies is presented in Table 6.

**Table 6:** Summary of predictive modeling studies assessing radiation-induced cardiac toxicity.

Study (year)	Cancer type	Modality/features used	Prediction target	Model type	Performance
Cai et al (2025)^112^	Esophageal	FDG-PET pre/post-RT (segmental SUV)	MACEs (MI, HF, death)	ΔSUV ratio + multivariable model	AUCs validated; significant basal SUV change
Chen et al (2019)^113^	NSCLC	Echo ΔGLS post-RT	All-cause mortality	ΔGLS threshold	AUC 0.784; ΔGLS ≥13.65% predictive
Cho et al (2022)^111^	NSCLC	Post-RT FDG-PET + dosimetry	Grade ≥2 cardiac events	SUV thresholds + Cox model	Max LV SUV HR: 2.1; mean heart dose HR: 3.6
Choi et al (2023)^108^	Breast	CT + dose + substructure auto-seg	Acute coronary events	Deep CNN + CAM	AUC 0.94 (CV); 0.83 (indep. test)
Choi et al (2024)^110^	Lung	Pre-RT FDG-PET/CT radiomics	Abnormal cardiac uptake (pre-existing risk)	Radiomics + ML	80%-92% accuracy (external validation)
Long et al (2025)^109^	Thoracic	Pre-RT CT radiomics (10 CV structures)	Cardiotoxicity (ΔLVEF, GLS)	OBM + SVM	Accuracy: up to 0.88 (LV), 0.84 (others)
Talebi et al (2025)^114^	Breast	Echo radiomics + dose + clinical	Cardiotoxicity at 6 months	ML w/FS + classifiers	AUC up to 0.97 (all features)

Abbreviations: AUC = area under the curve; CAM = class activation map; CNN = convolutional neural network; CV = cross-validation; Echo = echocardiography; FDG-PET = fluorodeoxyglucose positron emission tomography; GLS = global longitudinal strain; HF = heart failure; HR = hazard ratio; LVEF = left ventricular ejection fraction; LV = left ventricle; MACEs = major adverse cardiac events; MI = myocardial infarction; ML = machine learning; OBM = optimal biomarker model; PET = positron emission tomography; RT = radiotherapy; SUV = standardized uptake value; SVM = support vector machine.

## Discussion

Cardiac toxicity following RT is an increasingly recognized concern in survivors of breast, thoracic, and hematologic cancers.^115–118^ Imaging plays a critical role in detection, monitoring, and risk prediction of this delayed injury. Echocardiography, particularly GLS, remains the most accessible modality for serial assessment and has shown strong associations with subclinical dysfunction and future cardiac events. However, its limitations in spatial resolution and operator dependence constrain its ability to localize injury. CMR offers superior tissue characterization and has revealed spatially concordant fibrosis, edema, and inflammation in irradiated regions, although cost and contraindications limit widespread clinical use.

Other modalities offer complementary insights. PET imaging using [18F]-FDG can visualize metabolic changes indicative of inflammation or early remodeling, while SPECT imaging can detect perfusion defects and functional abnormalities. However, clinical integration of these techniques remains inconsistent. CT-based imaging, traditionally anatomical, has shown promise in detecting radiation-associated changes such as CAC and increased ECV. Building on these findings, newer techniques such as radiomics and photon-counting CT (PCCT) are being explored to capture vascular and structural changes with enhanced resolution.^119^^,^^120^ These developments support a future in which CT could serve dual oncologic and cardiac surveillance roles.

Despite these emerging approaches, several limitations persist. First, there is a lack of standardization in imaging protocols, timing of acquisition, and endpoints across studies. This variability makes it difficult to synthesize findings or translate them into guidelines. Second, most studies are retrospective, single-institutional, and underpowered for cardiac outcomes, which are often rare. Prospective imaging registries with long-term cardiac follow-up are urgently needed to advance the field.

Importantly, this review is limited to imaging-based studies, yet it is well recognized that radiation-induced cardiac toxicity arises from multifactorial mechanisms. Circulating biomarkers such as high-sensitivity troponin, NT-proBNP, and inflammatory cytokines have demonstrated prognostic value in identifying subclinical cardiac injury after RT.^121–123^ A future research agenda must prioritize multi-modal integration, combining imaging, dosimetry, serum biomarkers, and potentially genomic or proteomic data.

Looking forward, predictive modeling will play an increasingly important role. Recent studies leveraging machine learning and radiomics have demonstrated encouraging performance in forecasting cardiotoxicity based on imaging and dose features. To support clinical adoption, these models must first undergo rigorous external validation across diverse populations. While interpretability is also valuable—particularly in facilitating clinician trust and regulatory acceptance, it may be considered a secondary priority relative to validation. In parallel, standardization of cardiac substructure segmentation, dose metrics, and image-derived features will be essential for multi-institutional applicability.

Several recent and ongoing prospective trials have begun incorporating imaging endpoints to monitor RT-induced cardiotoxicity. For example, the RADCOMP (NCT02603341) trial compares proton versus photon therapy in patients with breast cancer, with a companion study (NCT04361240) assessing cardiovascular function using echocardiography and serum biomarkers. Other trials, such as CareBest (NCT03301389) and cardiac effects from RT by MRI (NCT04486573), utilize cardiac MRI to evaluate early changes in cardiac structure and function; however, the latter remains a small pilot study and may be underpowered to detect definitive cardiotoxicity endpoints. While these studies represent important progress, none are designed to directly compare imaging modalities. Given the financial, ethical, clinical and logistical constraints to conducting large-scale, head-to-head imaging comparisons, particularly the high cost and resource demands of advanced modalities like CMR, the optimal surveillance approach remains uncertain.

In the absence of comparative imaging trials, future solutions may emerge from computational modeling. Generative AI techniques could enable synthesis of one imaging modality from another, such as predicting CMR-derived fibrosis maps from echo or CT features, thereby bridging modality gaps without exposing patients to additional imaging. Digital twin frameworks, which simulate individual patient trajectories by integrating imaging, dosimetry, and clinical data, could offer a powerful tool for forecasting cardiotoxicity risk and guiding personalized surveillance or intervention strategies.^124^

In parallel, technical innovations in imaging hardware may further expand cardiotoxicity detection capabilities. PCCT represents a promising technological frontier with the potential to improve cardiac imaging resolution^125^ and reduce noise at lower radiation doses.^126^ Its integration into routine RT planning may enable concurrent oncologic and cardiac surveillance. On the therapeutic side, advanced modalities such as proton and particle therapy continue to offer theoretical advantages in cardiac sparing.^127^ While these technologies have gained momentum, robust prospective data on long-term cardiac outcomes remain limited.

Advanced imaging has enabled researchers to investigate the correlation between radiation-induced cardiac toxicity and radiation dose to the whole heart (eg, MHD). Given the varying dose sensitivities across different cardiac subregions, recent studies have begun leveraging advanced AI techniques to explore the relationship between cardiac toxicity and substructure-specific doses (eg, LAD).^128^^,^^129^ These efforts may facilitate a more comprehensive understanding of radiation-induced toxicity and support the development of cardiac substructure-sparing treatment plans, ultimately contributing to more precise and personalized radiotherapy.^130^

Building on this, cardiac-focused 4D CT techniques have emerged as a valuable tool for capturing cardiac and respiratory motion during RT planning, allowing for more accurate estimation of dose to moving cardiac substructures such as the LAD and left ventricle (LV).^131^^,^^132^ While standard 4D-CT used in thoracic RT primarily accounts for respiratory motion, emerging ECG-gated 4D cardiac CT techniques offer enhanced temporal resolution may support detailed cardiac dose mapping and functional assessment. Additionally, contrast-enhanced CT-derived metrics, including ECV fraction, may help identify subclinical myocardial injury.^133^^,^^134^ The integration of 4D cardiac CT into RT workflows not only supports motion-informed dose assessment but may also offer a noninvasive pathway for future toxicity surveillance, especially when combined with emerging reconstruction techniques and radiomics pipelines.

Ultimately, improving outcomes for patients at risk of radiation-induced cardiotoxicity will require a paradigm shift from late detection to early intervention. Imaging must be coupled with individual patient characteristics, including baseline cardiovascular risk, prior systemic therapies, and treatment intent, standardized across timepoints and patient populations, and used to guide both surveillance strategies and therapeutic decision-making. Cardio-oncology is shifting from reactive treatment toward risk-adapted, proactive survivorship, and imaging will be at the heart of such evolution.

## Conclusion

Cardiac toxicity is a potentially serious adverse effect of RT, particularly in survivors of breast, thoracic, and hematologic malignancies. This review synthesized evidence from over 78 studies employing diverse imaging modalities, including echocardiography, cardiac MRI, PET/SPECT, and cardiac CT to detect and characterize subclinical and clinical manifestations of radiation-induced cardiac injury. Across modalities, strain imaging and tissue characterization techniques have demonstrated utility in identifying early dysfunction and spatial dose-response relationships. Predictive modeling approaches integrating imaging, clinical, and dosimetric variables show promise for individualized risk assessment, but require further validation and standardization. Continued efforts to harmonize imaging protocols, design prospective trials with imaging endpoints, and develop explainable AI tools will be critical in translating these insights into routine cardio-oncology care.
